# Impact of Respiratory Infection and Chronic Comorbidities on Early Pediatric Antibiotic Dispensing in the United States

**DOI:** 10.1093/cid/ciac811

**Published:** 2022-10-05

**Authors:** Stephen M Kissler, Bill Wang, Ateev Mehrotra, Michael Barnett, Yonatan H Grad

**Affiliations:** Department of Immunology and Infectious Diseases, Harvard T. H. Chan School of Public Health, Boston, Massachusetts, USA; Department of Health Care Policy, Harvard Medical School, Boston, Massachusetts, USA; Department of Health Care Policy, Harvard Medical School, Boston, Massachusetts, USA; Department of Health Policy and Management, Harvard T. H. Chan School of Public Health, Boston, Massachusetts, USA; Division of General Internal Medicine and Primary Care, Brigham and Women's Hospital, Harvard Medical School, Boston, Massachusetts, USA; Department of Immunology and Infectious Diseases, Harvard T. H. Chan School of Public Health, Boston, Massachusetts, USA; Division of Infectious Diseases, Brigham and Women's Hospital, Harvard Medical School, Boston, Massachusetts, USA

**Keywords:** pediatric, antibiotic dispensing, respiratory infection, chronic comorbidity, medical claims

## Abstract

**Background:**

In the United States, children aged <5 years receive high volumes of antibiotics, which may contribute to antibiotic resistance. It has been unclear what role preventable illnesses and chronic comorbidities play in prompting antibiotic prescriptions.

**Methods:**

We conducted an observational study with a cohort of 124 759 children aged <5 years born in the United States between 2008 and 2013 with private medical insurance. Study outcomes included the cumulative number of antibiotic courses dispensed per child by age 5 and the proportion of children for whom at least 1 antibiotic course was dispensed by age 5. We identified which chronic medical conditions predicted whether a child would be among the top 20% of antibiotic recipients.

**Results:**

Children received a mean of 6.8 (95% confidence interval [CI]: 6.7–6.9) antibiotic courses by age 5, and 91% (95% CI: 90%–92%) of children had received at least 1 antibiotic course by age 5. Most antibiotic courses (71%; 95% CI: 70%–72%) were associated with respiratory infections. Presence of a pulmonary/respiratory, otologic, and/or immunological comorbidity substantially increase a child's odds of being in the top 20% of antibiotic recipients. Children with at least 1 of these conditions received a mean of 10.5 (95% CI: 10.4–10.6) antibiotic courses by age 5.

**Conclusions:**

Privately insured children in the United States receive many antibiotics early in life, largely due to respiratory infections. Antibiotic dispensing varies widely among children, with more antibiotics dispensed to children with pulmonary/respiratory, otologic, and/or immunological comorbidities.

The clinical effectiveness of antibiotics is increasingly threatened by antibiotic resistance, generated through evolutionary selective pressures exerted by antibiotic consumption [1]. To date, efforts to reduce antibiotic consumption have focused mainly on the elimination of inappropriate antibiotic prescriptions through physician outreach and education. Coinciding with these efforts, antibiotic prescribing in the United States has declined over the past few decades [2, 3]. However, only a minority of this decline was attributable to improved prescribing practices; most was driven by a concurrent decline in outpatient visit rates, especially for respiratory infections among children [4]. This suggests that primary disease prevention, for example, through vaccination or nonpharmaceutical interventions, may be an effective alternate strategy to further reduce antibiotic consumption [5, 6].

To establish a disease-prevention strategy aimed at reducing antibiotic consumption in a population, it is necessary to first quantify the baseline volume of antibiotic consumption, both overall and stratified by specific pathogens against which interventions might be deployed. Furthermore, since population-wide interventions (eg, universal vaccination) may be infeasible, it is important to identify which groups would yield maximal reductions in antibiotic consumption if targeted with a disease-prevention intervention. Children aged <5 years receive antibiotics at an especially high rate [7] and thus constitute a natural target population for such interventions. However, little is known about the correlates of high antibiotic use within this age group. Certain chronic comorbidities predispose children to higher rates of infection [8, 9], but the link between chronic comorbidities and antibiotic prescribing has not been adequately studied.

To address the evidence gap in pediatric antibiotic consumption and its link with chronic comorbidities, we used medical claims data from privately insured US children to estimate the cumulative number of antibiotics dispensed by age 5, the probability of receiving at least 1 antibiotic course by age 5, and the variation among individual children in the number of antibiotic courses received. Motivated by prior studies that highlighted the important role of respiratory infections in pediatric antibiotic consumption [4, 10] and by the possibility of preventing such infections through vaccines or nonpharmaceutical interventions, we separately measured antibiotic courses that were prompted by respiratory infections vs those prompted by other medical conditions. Finally, we identified a set of chronic conditions that predispose children to be in the top 20% of antibiotic consumers, highlighting a candidate target group for enhanced respiratory disease prevention.

## METHODS

### Study Sample and Data Sources

Data on dispensed antibiotics were obtained from pharmacy claims records aggregated in the IBM MarketScan database [11]. These data represent a convenience sample of 19.1–24.3 million individuals (5.9%–7.6% of the US population) with private medical insurance coverage, which varies based on insurance enrollment by month. Each pharmacy claim includes the fill date; the National Drug Code identifier; a drug class (Supplementary Table 1); and the patient's age, sex, state of residence, and metropolitan statistical area (MSA) [12] of residence.

The database also includes outpatient and inpatient claims with a primary diagnosis coded using the *International Classification of Diseases, Ninth Revision, Clinical Modification* (ICD9) or revision 10 (ICD10). We restricted the sample to children continuously enrolled through age 5 who were born between 1 January 2008 and 31 December 2013 with continuous representation in both the outpatient and pharmacy claims databases, following previous methods [13] (n = 124 759; Table 1, Supplementary Table 2).

**Table 1.** Summary of the Study Population

Category	N	%
Total	124 759	100
Sex		
ȃFemale	64 168	51.4
ȃMale	60 591	48.6
Birth year		
ȃ2008	22 060	17.7
ȃ2009	24 892	20.0
ȃ2010	21 560	17.3
ȃ2011	21 989	17.6
ȃ2012	18 438	14.8
ȃ2013	15 820	12.7

Number of individuals (N) and percent of the total cohort (%) by sex and birth year.

### Linking Outpatient Diagnoses to Antibiotic Courses

Dispensed antibiotic courses were linked to the most recent outpatient visit within 7 days, following previous methods [4, 14]. If a course was linked with an outpatient claim that listed a respiratory infection in either the first or second diagnosis field, then the course was deemed to be due to a respiratory infection. We restricted to the first 2 diagnosis fields because this was the maximum number of outpatient diagnoses available during the earliest years covered by the database (2008 and 2009). After 2010, up to 4 diagnoses were recorded. A respiratory infection was included in the third or fourth position in just 0.8% of the outpatient visit claims after 2010; of these, 76.7% also had a respiratory infection coded in the first or second position, so we anticipate that the bias introduced by including only the first 2 claims is small.

Respiratory infection diagnoses were identified by mapping ICD codes to Clinical Classification Software (CCS) codes [15] and extracting CCS codes consistent with sinusitis, pneumonia, influenza, tonsillitis, acute bronchitis, otitis media, and other unspecified upper respiratory infections (Supplementary Table 3). Use of CCS codes as an intermediary allowed us to use a common classification system despite the change from ICD9 to ICD10 that occurred during the study period. The list of respiratory infections was drawn from prior analyses on antibiotic prescribing in the United States [16].

### Assigning Chronic Conditions

We identified chronic medical conditions for each child in the cohort according to the Pediatric Medical Complexity Algorithm (PMCA) [17]. The algorithm maps ICD diagnoses that pertain to chronic disease to 1 of 19 body systems (eg, respiratory/pulmonary, musculoskeletal, immunological, genetic). We used the least conservative version of the algorithm to identify noncomplex chronic disease; that is, we tagged any individual with at least 1 outpatient visit in 1 of the PMCA chronic condition categories as having a chronic condition pertaining to that body system [12].

### Study Outcomes and Covariates

We estimated the mean cumulative number of antibiotic courses dispensed per child in the United States by age 5, the proportion of children who had received at least 1 antibiotic course by age 5, and the fraction of total antibiotic courses that were received by the top 20% of recipients. We stratified these estimates by the diagnosis that prompted them (respiratory infection vs other medical conditions). We also estimated the change in a child's odds of being in the top 20% of antibiotic consumers as predicted by presence of a chronic condition in any of the 19 body systems described by the PMCA. For children with chronic conditions in any body systems that yielded at least a 2-fold increase in the odds of being in the top 20% of antibiotic recipients, we again measured the mean cumulative number of antibiotic courses received and the proportion of children who received at least 1 antibiotic course by age 5.

### Statistical Analyses

To adjust for biased sampling, we weighted each claim according to the child's sex and state of residence. Weights were defined by the ratio of the number of children aged <5 years in that state/sex group in the US population according to the 2010 US census [18] and by the number of children in the MarketScan database in the same state/sex group (Supplementary Table 2). This follows the methods used to generate the weights included in the MarketScan database, which allow for national aggregation. We formulated our own weights to allow for finer scales of aggregation as well (eg, by MSA or by birth year).

Confidence intervals (CIs) for the estimated antibiotic fill rates were calculated as exact 95% CIs for Poisson means.

To assess the relationship between chronic medical conditions and high-volume antibiotic consumption, we conducted a categorical logistic regression to measure the change in log-odds of being in the top 20% of antibiotic consumers contributed by each chronic medical condition in the PMCA. We used the following regression model:

$$y_{i}=\beta_{0}+\overset{n}{\underset{\;j=1}{\sum }}\;\beta_{j}I_{ij}+\epsilon_{i}$$

where *y*_*i*_ is the log-odds that individual *i* is in the top 20% of antibiotic consumers, *I*_*ij*_ is an indicator that is 1 if individual *i* has chronic condition *j* and 0 otherwise, and ε_*i*_ is an error term. The sum is overall *n* = 19 body systems in the PMCA, and the β_*j*_ terms are coefficients measuring the relationship between chronic conditions in each system and the odds of being in the top 20% of antibiotic consumers. The coefficient *β*_0_ is an intercept term that captures the baseline log-odds of being in the top 20% of antibiotic consumers. We estimated the coefficients using the *glm* function in R version 4.1.2 [19]. We report estimates and their *P* values according to a 2-tailed *t*-test. Full analysis code is available online [20].

This study was a secondary analysis of data collected for other purposes and was not considered human subjects research and not reviewed by an institutional review board.

## RESULTS

**Figure 1.**
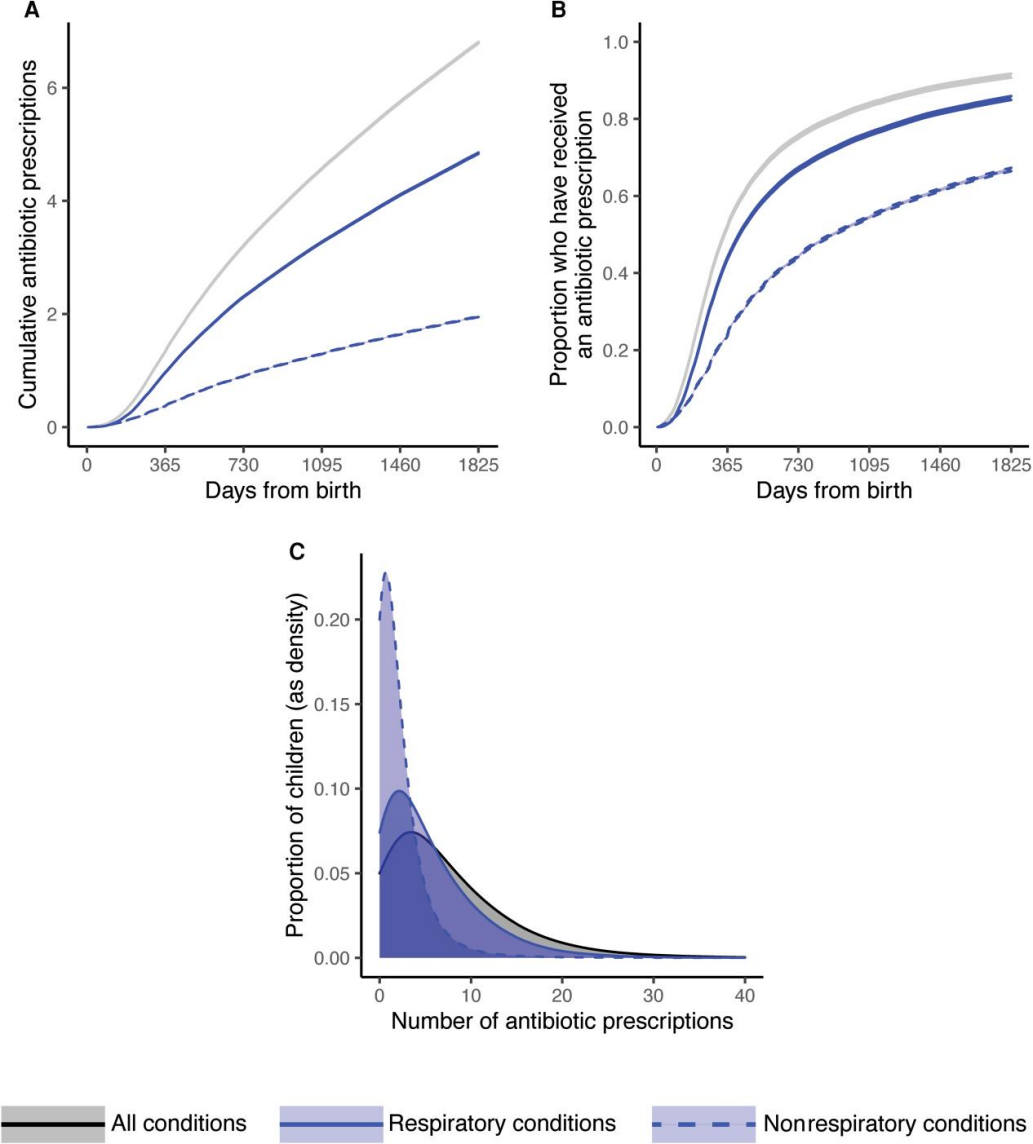
Patterns in dispensed antibiotics to children aged <5 years in the United States for respiratory and nonrespiratory conditions. *A*, Mean cumulative number of antibiotic courses received per child between birth and age 5. *B*, Proportion of children who have received at least 1 antibiotic course between birth and age 5. *C*, Histograms of the number of antibiotics received per child by age 5, depicting the variation in number of antibiotic courses received across children. In panels (*A*) and (*B*), lines depict means and bands depict 95% confidence intervals according to a 2-tailed *t* test for the mean. Colors correspond as follows: all conditions, black, solid (upper lines in (*A*) and (*B*), right-most distribution in (*C*)); respiratory conditions, blue, solid (middle lines in (*A*) and (*B*), middle distribution in (*C*)); nonrespiratory conditions, blue, dashed (lower lines in (*A*) and (*B*), left-most distribution in (*C*)).

Children in the cohort received a mean of 6.8 (95% CI: 6.7–6.9) antibiotic courses by age 5 (Figure 1*A*). The mean antibiotic dispensing rate varied by age, beginning at 0.60 courses per child per year (95% CI: .59–.61) between birth and 6 months of age (180 days), then increasing to 1.9 courses per child per year (95% CI: 1.8–2.0) between 6 months (180 days) and 2 years of age (730 days), before slowing again to 1.2 courses per child per year (95% CI: 1.1–1.3) between age 2 (730 days) and age 5 (1825 days). Most antibiotic courses (71%; 95% CI: 70%–72%, or 4.8 courses, 95% CI: 4.7–4.9) were associated with respiratory infections.

By age 5, 91% (95% CI: 90%–92%) of children had received at least 1 antibiotic course for any reason, and 85% (95% CI: 84%–86%) had received at least 1 antibiotic course for a respiratory infection (Figure 1*B*).

Children varied widely in the number of antibiotic courses received (Figure 1*C*). The top 20% of antibiotic recipients (ie, those with the most courses received by age 5) accounted for 52% of all dispensed antibiotic courses in this cohort.

The presence of an underlying medical condition generally increased the odds that a child would be among the top 20% of antibiotic recipients (Table 2). Pulmonary/respiratory conditions (odds ratio [OR], 2.64; *P* < .0001), otologic conditions (OR, 2.62; *P* < .0001), and immunological conditions (OR, 2.53; *P* < .0001) were associated with the greatest increase in the odds of being in this highest-consumption group. The most common CCS codes that marked a pulmonary/respiratory, otologic, or immunological condition were related to asthma, general immunity disorders, and hearing loss, respectively (Supplementary Table 4).

**Table 2.** Odds Ratios of Being in the Top 20% of Antibiotic Recipients for Children With vs Without Various Chronic Conditions

Body System	Odds Ratio	*P* Value	Number With Condition (%)	Percentage of Top 20% of Antibiotic Recipients With Condition
Pulmonary/respiratory	2.64	< .0001	22 786 (18.3)	7.0
Otologic	2.62	< .0001	2240 (1.8)	0.8
Immunological	2.53	< .0001	1435 (1.2)	0.6
Renal	1.96	< .0001	1315 (1.1)	0.4
Gastrointestinal	1.52	< .0001	2646 (2.1)	0.8
Neurological	1.36	< .0001	2985 (2.4)	0.8
Genitourinary	1.32	< .0001	11 118 (8.9)	2.7
Hematological	1.30	< .0001	1217 (1)	0.3
Cardiac	1.22	< .0001	2907 (2.3)	0.7
Endocrinological	1.21	< .01	1174 (0.9)	0.3
Metabolic	1.20	< .0001	5413 (4.3)	1.3
Dermatological	1.10	< .001	8522 (6.8)	1.8
Genetic	1.05	N.S.	341 (0.3)	0.1
Musculoskeletal	1.05	N.S.	1208 (1)	0.4
Craniofacial	1.05	N.S.	8969 (7.2)	1.8
Ophthalmological	1.02	N.S.	11 341 (9.1)	2.4
Mental health	0.98	N.S.	431 (0.3)	0.1
Malignancy	0.92	N.S.	558 (0.4)	0.1
Otolaryngological	0.87	N.S.	308 (0.2)	0.1

Abbreviation: N.S., not significant.

*P* values correspond to 2-tailed *t-*tests that the log-odds ratio differs from 0. Note that individuals may fall into more than 1 category if they have multiple chronic conditions.

**Figure 2.**
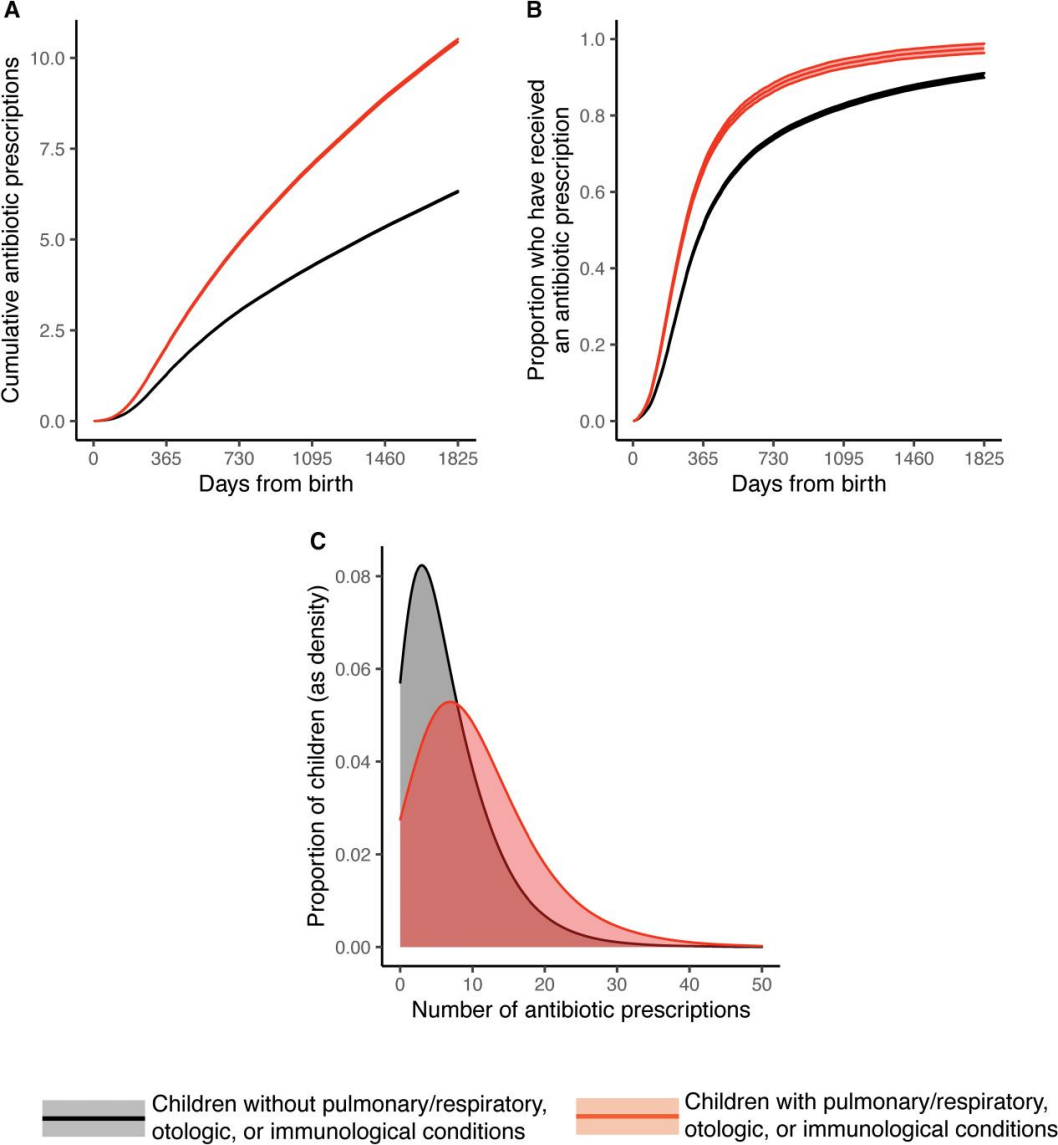
Patterns in dispensed antibiotics for children with and without pulmonary/respiratory, otologic, or immunological conditions. *A*, Mean cumulative number of antibiotic courses received per child between birth and age 5 years. *B*, Proportion of children who have received at least 1 antibiotic course between birth and age 5. *C*, Histograms of the number of antibiotics received per child by age 5, depicting the variation in number of antibiotic courses received across children. In panels (*A*) and (*B*), lines depict means and bands depict 95% confidence intervals according to a 2-tailed *t*-test for the mean. Colors correspond as follows: those without a pulmonary/respiratory, otologic, or immunological condition, black (lower lines in (*A*) and (*B*), left-most distribution in (*C*)); those with a pulmonary/respiratory, otologic, or immunological condition, red (upper lines in (*A*) and (*B*), right-most distribution in (*C*)).

Children with a chronic pulmonary/respiratory, otologic, or immunological condition received more antibiotic courses (10.5; 95% CI: 10.4–10.6) by age 5 than those without any of these conditions (6.3; 95% CI: 6.2–6.4; Figure 2*A*). Children with a chronic pulmonary/respiratory, otologic, or immunological condition also received their first antibiotic course earlier in life: by age 1 year, 66% of children (95% CI: 65%–67%) with at least 1 such condition had received at least 1 antibiotic course vs 51% (95% CI: 50%–52%) of children without any such conditions (Figure 2*B*). By age 5, 98% of children (95% CI: 96%–99%) with at least 1 such condition had received at least 1 antibiotic course vs 90% (95% CI: 89%–91%) of children without any such conditions (Figure 2*B*).

## DISCUSSION

Antibiotic dispensing for children aged <5 years in the United States is frequent (6.8 courses per child on average) and widespread (91% of children receive at least 1 antibiotic by age 5). Most (71%; 95% CI: 70%–72%) of these antibiotic courses are associated with respiratory infections. Presence of a chronic pulmonary/respiratory, otologic, or immunological condition more than double a child's risk of being among the top 20% of antibiotic recipients. Reductions in antibiotic use in this youngest age group may be possible by reducing the incidence of respiratory illnesses through vaccination or other interventions, especially among those with select chronic conditions. Such efforts should occur alongside other efforts to improve antibiotic stewardship, including improving the accuracy of diagnosis and increasing patient and physician awareness about the proper use of antibiotics.

Our results are broadly consistent with prior studies that have measured cumulative antibiotic use in children in other high-income countries and, within the United States, in more geographically restricted cohorts. In New Zealand, children received a mean of 8 antibiotic prescriptions before age 5, and 97% of children received at least 1 antibiotic by age 5 [21]. In a Philadelphia cohort, 69% of children received at least 1 antibiotic course before age 2 for a mean of 2.3 antibiotic courses per child, in line with our findings [22].

The coronavirus disease 2019 (COVID-19) pandemic has substantially altered the transmission of and care-seeking patterns for respiratory pathogens [23–25]. The timing and intensity of respiratory syncytial virus [24], influenza [23], and *Streptococcus pneumoniae* [26] cases in the population have undergone dramatic shifts, most likely due to the widespread adoption of nonpharmaceutical interventions to interrupt the spread of severe acute respiratory syndrome coronavirus 2 (SARS-CoV-2). Because of this and because of the introduction of SARS-CoV-2 as a novel respiratory pathogen, future rates of pediatric antibiotic consumption may differ from those reported here. We anticipate that COVID-19 will exacerbate the differences in antibiotic use between those with and without key chronic conditions, since COVID-19 leads to more severe disease in those with chronic comorbidities and severe disease increases the risk of secondary bacterial infection [27]. This study also took place during a period when antibiotic dispensing rates were declining [2, 3] (Supplementary Figure 1), so broad-scale temporal shifts in antibiotic dispensing rates should be taken into account when generalizing these findings to current or future antibiotic use patterns [28].

Limitations to this study include possible bias introduced by convenience sampling and the exclusion of individuals without insurance or with Medicare or Medicaid. The MarketScan database covers only around 10% of privately insured individuals in the United States, and our analysis covers a further restricted cohort that was continuously represented in the data for 5 years. The study sample was likely to exclude the dependents of individuals who frequently change employers (and thus insurance providers), since these were less likely to be represented continuously for 5 years in the data. Another limitation is that we were unable to directly observe the indication for prescribing. We inferred the indication by looking at associated diagnosis codes with recent visits proximal to the filled prescription, but this may misclassify some antibiotic courses. It is also possible that some antibiotics were linked with respiratory conditions but were not prescribed primarily for the respiratory condition; for example, if a patient was diagnosed with both bronchitis and a urinary tract infection, an antibiotic may have been given for the urinary tract infection but would have still been linked in this analysis with the respiratory condition. Additionally, we could not distinguish between appropriate and inappropriate prescribing of antibiotics or nonantibiotics. We also could not assess antibiotics that were administered directly at the point of care, so our estimates may underrepresent overall outpatient antibiotic use.

In conclusion, children in the United States are exposed to antibiotics frequently and at an early age. Reducing antibiotic use remains a public health priority. Reducing rates of respiratory illness, through vaccination or other means, may be an effective way to achieve reductions in early pediatric antibiotic use, especially if these interventions are targeted to children with chronic conditions that predispose them to frequent receipt of antibiotics.

## Supplementary Data

Supplementary materials are available at *Clinical Infectious Diseases* online. Consisting of data provided by the authors to benefit the reader, the posted materials are not copyedited and are the sole responsibility of the authors, so questions or comments should be addressed to the corresponding author.

## Supplementary Material

ciac811_Supplementary_Data

## Notes

***Author contributions.*** S. M. K. conceptualized and designed the study, conducted the statistical analysis, drafted the initial manuscript, and reviewed and revised the manuscript. B. W. conducted the statistical analysis and reviewed and revised the manuscript. A. M., M. B., and Y. H. G. conceptualized and designed the study, secured data access, reviewed and revised the manuscript, and oversaw the work. All authors approved the final manuscript as submitted and agree to be accountable for all aspects of the work.

***Financial support*.** Y. H. G. reports support from the Wellcome Trust and CDC contract 200-2016-91779 (to institution).
