# Supplementary material for: Impact of Respiratory Infection and Chronic Comorbidities on Early Pediatric Antibiotic Dispensing in the United States
Source: Clin Infect Dis. 2022 Oct 5;76(3):382–8. doi: 10.1093/cid/ciac811 (PMC9907510; doi:10.1093/cid/ciac811)
Supplement: ciac811_Supplementary_Data [file ciac811_supplementary_data.docx]

**Supplementary Text.**

Temporal analysis. To assess temporal variation in antibiotic dispensing, we generated estimates of the total number of antibiotic courses received by age 5 stratified by birth year.

Antibiotic dispensing rates declined steadily across the years of this study, with children born in 2008 receiving an average of 7.5 antibiotic courses (95% CI 7.4, 7.6) by age 5 and children born in 2013 receiving an average of 6.1 antibiotic courses (95% CI 6.0, 6.2) by age 5 (**Supplementary Figure 1**).

Geographic analysis. To assess geographic variation in antibiotic dispensing, we measured dispensed antibiotic courses per child by census region (Northeast, South, Mid/Mountain West, and Pacific West; **Supplementary Table 4**) and by metropolitan statistical area (MSA),^14^ a designation assigned by the US Census Bureau that roughly corresponds to a city. For the MSA-level analysis, we excluded MSAs that had fewer than 100 cohort members. These remaining 174 MSAs included 82% (*n =* 102,161) of the study population.

Cumulative antibiotic courses by age 5 for both respiratory infections and other conditions were higher in MSAs in the South (respiratory infections: 5.7 (95% CI 5.6, 5.8); other conditions: 2.3 (95% CI 2.2, 2.4)) than in the rest of the country, such as the Pacific West (respiratory infections: 3.4 (95% CI 3.2, 3.5); other conditions: 1.5 (95% CI 1.3, 1.6)) (**Supplementary Figure 2A**). The penetrance of antibiotic use was also higher in the South. For example, the proportion of children who received at least one antibiotic course for a respiratory infection by age 5 was 88% (95% CI 87%, 89%) in the South *vs.* 79% (95% CI 78%, 80%) in the Pacific West (**Supplementary Figure 2B**).

Venues of antibiotic prescribing. Each outpatient claim was tagged with the venue type. Based on these tags, we found that 87.9% of antibiotic courses were associated with an outpatient office visit, 4.6% with a visit to an outpatient hospital, 3.3% with a visit to an urgent care facility, 1.4% to an emergency room visit, and 1.3% with a visit to an independent laboratory. The remaining venues accounted for fewer than 1% of visits each.


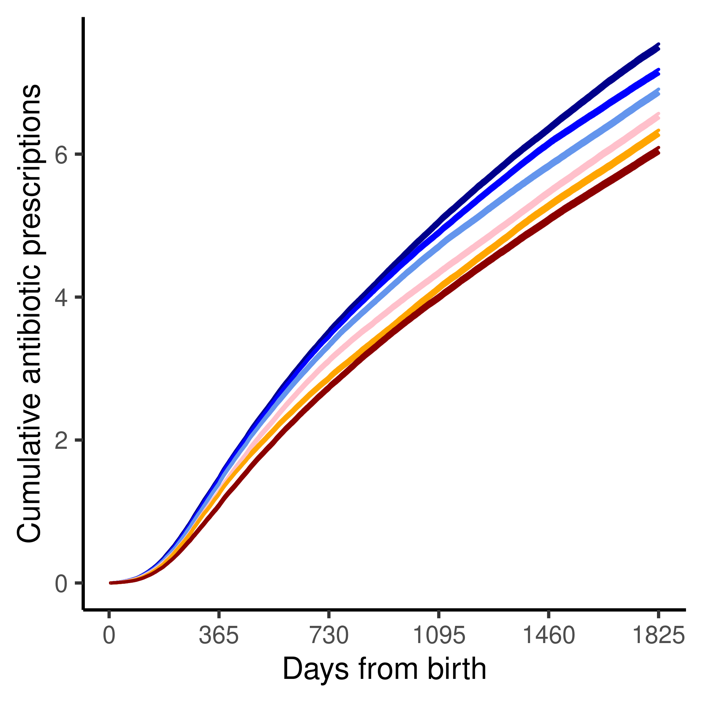


Birth year

2013

2012

2011

2010

2009

2008

Supplementary Figure 1. Cumulative antibiotic courses received by children from birth to age 5 stratified by birth year. Lines depict means and bands depict 95% confidence intervals according to a two-tailed *t*-test for the mean.


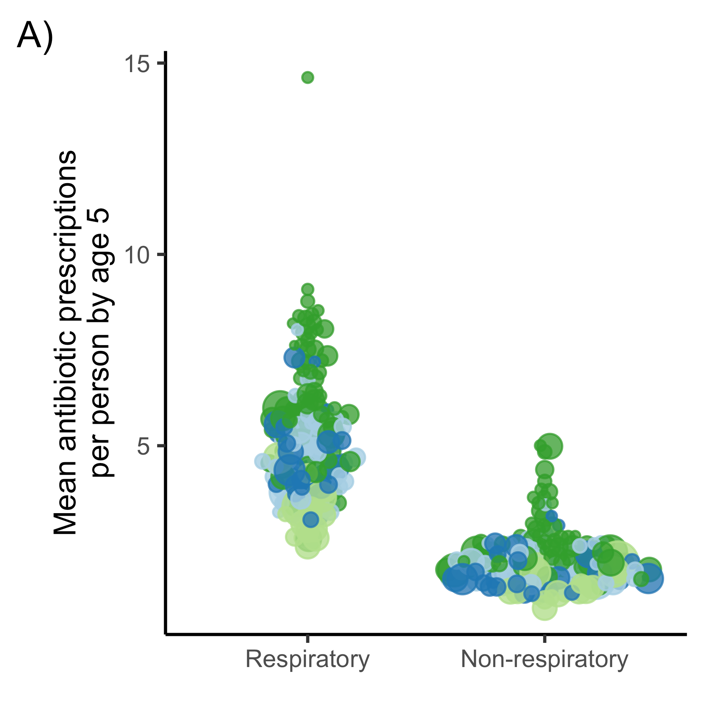

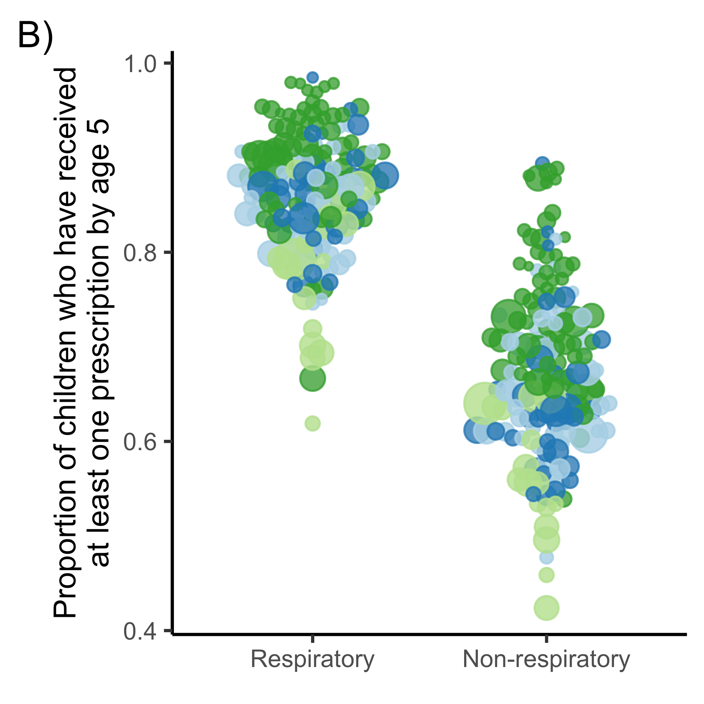


Population size


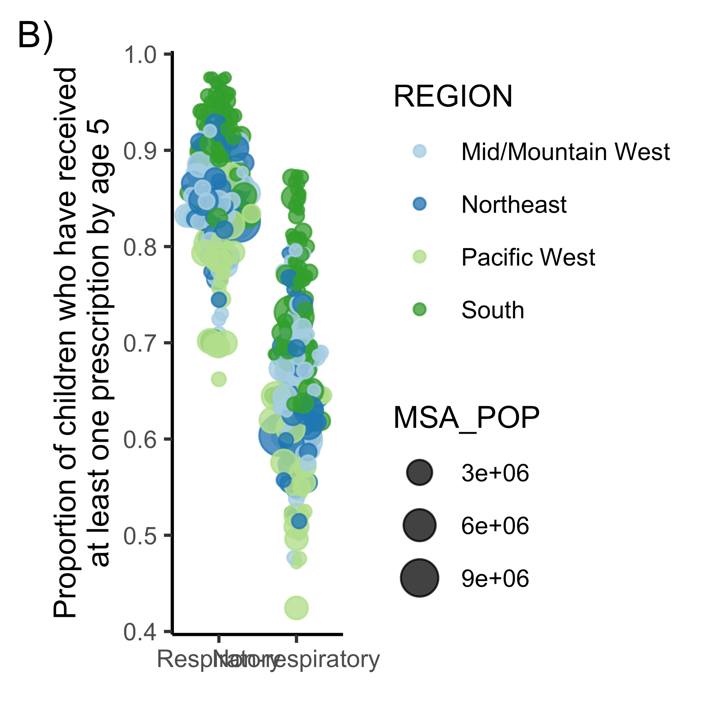

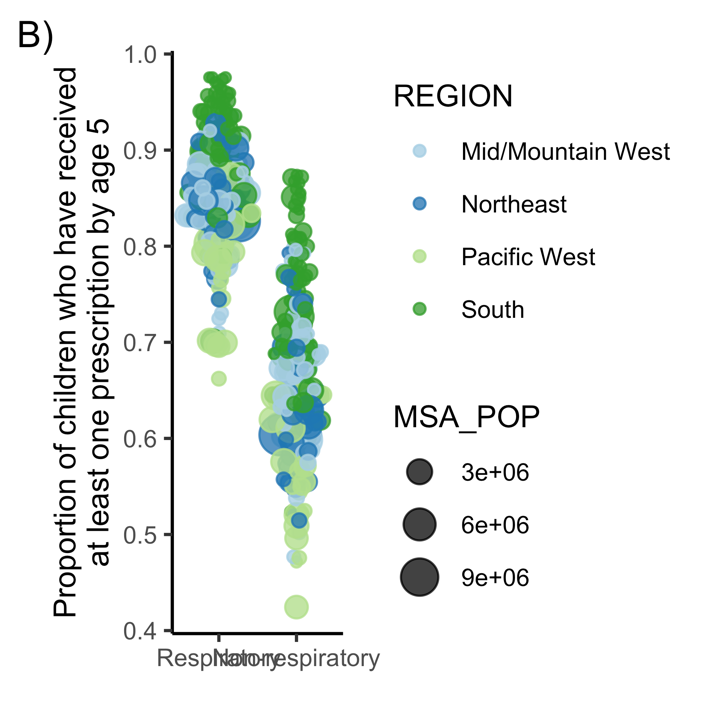


Supplementary Figure 2. Geographic distribution of antibiotic use for children under age 5 in the United States by Metropolitan Statistical Area and medical condition. Bee swarm plots depicting the distribution of (A) the mean cumulative number of antibiotic courses filled per child between birth and age 5 and (B) the proportion of children who have received at least one antibiotic course between birth and age 5 for 174 of the largest Metropolitan Statistical Areas (MSAs) in the US, separated by respiratory and non-respiratory indications. Points represent Metropolitan Statistical Areas (MSAs), point size is proportional to the MSA’s population size, and colors represent the geographic region where the MSA is located.

Supplementary Table 1. List of therapeutic classes used to specify “antibiotics”. A full list of the antibiotic classes, names, and NDC codes are provided as a data file (abxlist.csv) in the online code repository at <https://github.com/skissler/PediatricPrescribing_Chronic>.

| **Therpeutic Class** |
| --- |
| Antibiot, Penicillins |
| Antibiot, Aminoglycosides |
| Antibiotics, Misc |
| Antibiot, Cephalosporin & Rel. |
| Antibiot, B-Lactam Antibiotics |
| Antibiot, Erythromycn&Macrolid |
| Anti-Infectives, Misc |
| Antibiot, Tetracyclines |
| Antibiot, Chloramphenicol&Comb |
| Urinary Anti-Infectives, NEC |

Supplementary Table 2. Characteristics of the study population. Number of individuals (*N*), reference population sizes and population fractions from the 2010 US census, and weights applied to each claim, equal to the 2010 US Census population fraction divided by the number of individuals *N*.

| **State** | **Sex** | ***N*** | **Population size, 2010 US Census** | **Population fraction,  2010 US Census** | **Weight (x 10^-5^)** |
| --- | --- | --- | --- | --- | --- |
| Alabama | F | 258 | 148372 | 0.0074 | 2.9 |
| Alabama | M | 317 | 154287 | 0.0077 | 2.4 |
| Alaska | F | 65 | 24646 | 0.0012 | 1.9 |
| Alaska | M | 72 | 26714 | 0.0013 | 1.9 |
| Arizona | F | 1144 | 226542 | 0.0113 | 1 |
| Arizona | M | 1215 | 236518 | 0.0118 | 1 |
| Arkansas | F | 273 | 95376 | 0.0048 | 1.7 |
| Arkansas | M | 262 | 100110 | 0.005 | 1.9 |
| California | F | 1589 | 1244216 | 0.0621 | 3.9 |
| California | M | 1708 | 1300849 | 0.0649 | 3.8 |
| Colorado | F | 734 | 166096 | 0.0083 | 1.1 |
| Colorado | M | 813 | 173902 | 0.0087 | 1.1 |
| Connecticut | F | 840 | 100161 | 0.005 | 0.6 |
| Connecticut | M | 873 | 105318 | 0.0053 | 0.6 |
| Delaware | F | 651 | 27534 | 0.0014 | 0.2 |
| Delaware | M | 657 | 28476 | 0.0014 | 0.2 |
| Florida | F | 3447 | 528479 | 0.0264 | 0.8 |
| Florida | M | 3672 | 552027 | 0.0275 | 0.7 |
| Georgia | F | 6443 | 336710 | 0.0168 | 0.3 |
| Georgia | M | 6846 | 351811 | 0.0176 | 0.3 |
| Idaho | F | 181 | 58198 | 0.0029 | 1.6 |
| Idaho | M | 192 | 60968 | 0.003 | 1.6 |
| Illinois | F | 3014 | 413907 | 0.0206 | 0.7 |
| Illinois | M | 3096 | 431211 | 0.0215 | 0.7 |
| Indiana | F | 1269 | 212592 | 0.0106 | 0.8 |
| Indiana | M | 1315 | 221612 | 0.0111 | 0.8 |
| Iowa | F | 569 | 96793 | 0.0048 | 0.8 |
| Iowa | M | 619 | 101153 | 0.005 | 0.8 |
| Kansas | F | 538 | 98435 | 0.0049 | 0.9 |
| Kansas | M | 594 | 100765 | 0.005 | 0.8 |
| Kentucky | F | 748 | 136071 | 0.0068 | 0.9 |
| Kentucky | M | 735 | 142230 | 0.0071 | 1 |
| Louisiana | F | 497 | 149242 | 0.0074 | 1.5 |
| Louisiana | M | 561 | 155698 | 0.0078 | 1.4 |
| Maine | F | 171 | 33622 | 0.0017 | 1 |
| Maine | M | 155 | 36243 | 0.0018 | 1.2 |
| Maryland | F | 528 | 178868 | 0.0089 | 1.7 |
| Maryland | M | 531 | 186225 | 0.0093 | 1.7 |
| Massachusetts | F | 2754 | 180330 | 0.009 | 0.3 |
| Massachusetts | M | 2837 | 187996 | 0.0094 | 0.3 |
| Michigan | F | 3399 | 300463 | 0.015 | 0.4 |
| Michigan | M | 3436 | 314019 | 0.0157 | 0.5 |
| Minnesota | F | 687 | 173098 | 0.0086 | 1.3 |
| Minnesota | M | 778 | 179512 | 0.009 | 1.2 |
| Mississippi | F | 827 | 101976 | 0.0051 | 0.6 |
| Mississippi | M | 857 | 106515 | 0.0053 | 0.6 |
| Missouri | F | 1707 | 188870 | 0.0094 | 0.6 |
| Missouri | M | 1903 | 197907 | 0.0099 | 0.5 |
| Montana | F | 59 | 29191 | 0.0015 | 2.5 |
| Montana | M | 81 | 30531 | 0.0015 | 1.9 |
| Nebraska | F | 320 | 63576 | 0.0032 | 1 |
| Nebraska | M | 347 | 65834 | 0.0033 | 0.9 |
| Nevada | F | 301 | 91863 | 0.0046 | 1.5 |
| Nevada | M | 286 | 96732 | 0.0048 | 1.7 |
| New Hampshire | F | 347 | 35525 | 0.0018 | 0.5 |
| New Hampshire | M | 404 | 36772 | 0.0018 | 0.5 |
| New Jersey | F | 2332 | 267716 | 0.0134 | 0.6 |
| New Jersey | M | 2571 | 279434 | 0.0139 | 0.5 |
| New Mexico | F | 315 | 70353 | 0.0035 | 1.1 |
| New Mexico | M | 356 | 72947 | 0.0036 | 1 |
| New York | F | 2680 | 566415 | 0.0283 | 1.1 |
| New York | M | 2923 | 592250 | 0.0295 | 1 |
| North Carolina | F | 2072 | 303237 | 0.0151 | 0.7 |
| North Carolina | M | 2195 | 318634 | 0.0159 | 0.7 |
| North Dakota | F | 51 | 20473 | 0.001 | 2 |
| North Dakota | M | 54 | 21319 | 0.0011 | 2 |
| Ohio | F | 3910 | 355298 | 0.0177 | 0.5 |
| Ohio | M | 4154 | 369367 | 0.0184 | 0.4 |
| Oklahoma | F | 481 | 125707 | 0.0063 | 1.3 |
| Oklahoma | M | 453 | 130917 | 0.0065 | 1.4 |
| Oregon | F | 809 | 114208 | 0.0057 | 0.7 |
| Oregon | M | 873 | 119993 | 0.006 | 0.7 |
| Pennsylvania | F | 1385 | 355520 | 0.0177 | 1.3 |
| Pennsylvania | M | 1450 | 371755 | 0.0185 | 1.3 |
| Rhode Island | F | 138 | 28772 | 0.0014 | 1 |
| Rhode Island | M | 185 | 30148 | 0.0015 | 0.8 |
| South Carolina | F | -- | 145046 | 0.0072 | -- |
| South Carolina | M | -- | 150860 | 0.0075 | -- |
| South Dakota | F | 69 | 27395 | 0.0014 | 2 |
| South Dakota | M | 64 | 29099 | 0.0015 | 2.3 |
| Tennessee | F | 1409 | 197994 | 0.0099 | 0.7 |
| Tennessee | M | 1466 | 205473 | 0.0103 | 0.7 |
| Texas | F | 6215 | 931442 | 0.0465 | 0.7 |
| Texas | M | 6651 | 972112 | 0.0485 | 0.7 |
| Utah | F | 622 | 124041 | 0.0062 | 1 |
| Utah | M | 604 | 130971 | 0.0065 | 1.1 |
| Vermont | F | 33 | 15557 | 0.0008 | 2.4 |
| Vermont | M | 38 | 16698 | 0.0008 | 2.2 |
| Virginia | F | 947 | 247543 | 0.0123 | 1.3 |
| Virginia | M | 1077 | 258706 | 0.0129 | 1.2 |
| Washington | F | 596 | 208047 | 0.0104 | 1.7 |
| Washington | M | 644 | 218403 | 0.0109 | 1.7 |
| Washington DC | F | 14 | 15959 | 0.0008 | 5.7 |
| Washington DC | M | 20 | 16463 | 0.0008 | 4.1 |
| West Virginia | F | 111 | 50532 | 0.0025 | 2.3 |
| West Virginia | M | 121 | 53226 | 0.0027 | 2.2 |
| Wisconsin | F | 1047 | 173266 | 0.0086 | 0.8 |
| Wisconsin | M | 1020 | 181588 | 0.0091 | 0.9 |
| Wyoming | F | 48 | 18153 | 0.0009 | 1.9 |
| Wyoming | M | 58 | 19115 | 0.001 | 1.6 |

Supplementary Table 3. Clinical classification software (CCS) codes for respiratory infections. Columns list the CCS code for the ICD9 (2015) and ICD10 (2021) revisions. A full list of CCS codes with associated ICD diagnosis codes and labels are provided as a data file (ccs_map_desc.csv) in the online code repository at <https://github.com/skissler/PediatricPrescribing_Chronic>.

| Condition | CCS9 |  | CCS10 |
| --- | --- | --- | --- |
| Sinusitis | – |  | RSP001 |
| Pneumonia | 122 |  | RSP002 |
| Influenza | 123 |  | RSP003 |
| Tonsillitis | 124 |  | RSP004 |
| Bronchitis (acute) | 125 |  | RSP005 |
| Upper respiratory infection (other) | 126 | (Includes Sinusitis) | RSP006 |
| Otitis media | 92 |  | EAR001 |

Supplementary Table 4. Top three CCS conditions associated with chronic immunological, otologic, and pulmonary/respiratory conditions as defined by the PMCA. Counts are stratified by the body system and the ICD version used to code the diagnoses. Note that in the ICD-10 CCS mapping scheme, hearing loss is included within “Other ear dx”.

| **Body System** | **ICD version** | **CCS label** | **Number of visits** | **% of visits with diagnoses in this body system and ICD version with this CCS label** |
| --- | --- | --- | --- | --- |
| Immunological | 10 | Immunity disorders | 593 | 40.4 |
|  |  | Juvenile arthritis | 332 | 22.6 |
|  |  | Systemic lupus erythematosus | 210 | 14.3 |
|  | 9 | Immunity dx | 2860 | 46.5 |
|  |  | Rheum arth | 1143 | 18.6 |
|  |  | Wht blood dx | 793 | 12.9 |
| Otologic | 10 | Hearing loss | 1941 | 77.7 |
|  |  | Diseases of middle ear and mastoid | 269 | 10.8 |
|  |  | Other specified and unspecified disorders | 143 | 5.7 |
|  | 9 | Other ear dx | 1922 | 85.0 |
|  |  | Dizziness | 216 | 9.6 |
|  |  | Otitis media | 122 | 5.4 |
| Pulmonary/  Respiratory | 10 | Asthma | 14385 | 82.0 |
|  |  | Sleep wake disorders | 2209 | 12.6 |
|  |  | Respiratory perinatal condition | 406 | 2.3 |
|  | 9 | Asthma | 60821 | 77.0 |
|  |  | Ot perint dx | 4996 | 6.3 |
|  |  | Unclassified | 4686 | 5.9 |

**Supplementary Table 5.** **Regions with constituent HHS categories and states.** HHS categories^32^ consist of groupings of states that are further grouped into the four regions in our analysis.

| **Region** | **HHS categories** | **States** |
| --- | --- | --- |
| Northeast | 1, 2, 3 | Connecticut, Delaware, Maine, Maryland, Massachusetts, New Hampshire, New Jersey, New York, Pennsylvania, Rhode Island, Vermont, Virginia, Washington DC, West Virginia |
| South | 4, 6 | Alabama, Arkansas, Florida, Georgia, Kentucky, Louisiana, Mississippi, New Mexico, North Carolina, Oklahoma, South Carolina, Tennessee, Texas |
| Mid/Mountain West | 5, 7, 8 | Colorado, Illinois, Indiana, Iowa, Kansas, Michigan, Minnesota, Missouri, Montana, Nebraska, North Dakota, Ohio, South Dakota, Utah, Wisconsin, Wyoming |
| Pacific West | 9, 10 | Alaska, Arizona, California, Hawaii, Idaho, Nevada, Oregon, Washington |
